# Finite Element Analysis of 3D-Printed Gears: Evaluating Mechanical Behaviour Through Numerical Modelling

**DOI:** 10.3390/ma18194530

**Published:** 2025-09-29

**Authors:** Costin Nicolae Ilincă, Ibrahim Naim Ramadan, Adrian Neacșa, Marius Gabriel Petrescu, Eugen Victor Laudacescu

**Affiliations:** Department of Mechanical Engineering, Petroleum-Gas University of Ploiesti, 100680 Ploiesti, Romania; icostin@upg-ploiesti.ro (C.N.I.); pmarius@upg-ploiesti.ro (M.G.P.)

**Keywords:** polyamide 6 (PA6), 3D printing, heat treatments, annealing, mechanical properties, polymer gears, finite element analyses, numerical approach, stress distributions

## Abstract

In the course of the 3D printing process, the occurrence of imperfect structures is attributable to the rapid cooling of molten polymer. In this study, gears were manufactured from PA6 using a dedicated 3D printer, and their performance was analyzed using finite element analysis (FEA), validated by wear tests. A subset of the gears was subjected to annealing heat treatments to investigate their influence on the behavior of the material. The novelty of this study lies in the correlation of the effects induced by heat treatment with the stress distribution, wear, and service life of 3D-printed gears. This provides useful information for optimizing polymer gears for engineering applications. This study’s novelty lies in highlighting the influence of heat treatments on wear behaviour and mechanical stress factors, offering new insights into the optimisation of 3D-printed polymer gears.

## 1. Introduction

This comprehensive introduction sets the stage for a detailed exploration of the impact of heat treatments on the mechanical properties of 3D-printed PA6 polymer gears, providing a roadmap for readers to navigate through the intricacies of the research.

Additive manufacturing (AM) has transformed gear fabrication, offering design flexibility, rapid prototyping, and the potential to replace conventional metallic gears in engineering-specific applications. Among printable polymers, Polyamide 6 (PA6) is notable for its good balance of strength, toughness, and tribological properties [1,2,3].

However, several critical research gaps in this field remain:

- The materials selected must have physicochemical, mechanical and technological properties that meet the requirements of the application;
- The impact of 3D printing parameters on gear dimensional accuracy and precision has not been systematically studied;
- The influence of post-printing heat treatments on dimensional stability and long-term wear remains unclear;
- Most finite element analyses of polymer gears assume isotropic properties, overlooking anisotropy from layered manufacturing;
- Current simulations rarely consider dynamic loads or extreme temperatures that reflect real-world applications.

The novelty of this work lies in combining experimental and numerical approaches to evaluate the mechanical behaviour of 3D-printed PA6 gears before and after heat treatment. The research emphasises dimensional stability, wear resistance, and bending/contact stress under service-like conditions, bridging the knowledge gap between traditional gear studies (primarily metals and injection-moulded plastics) and additive-manufactured polymer gears.

PA6 stands out for its superior mechanical properties among the many materials used in this process. However, the rapid cooling associated with 3D printing of PA6 can lead to structural imperfections, and further research into optimisation techniques is required. With a focus on achieving improved mechanical strength, this research aims to elucidate the effect of heat treatments on the mechanical properties of 3D-printed PA6.

Expanding research efforts to include plastic gears is essential for enhancing our understanding of how these materials perform under different operational conditions. This broader investigation can contribute to refining plastic gear designs and developing specific guidelines and methodologies for assessing and optimizing their performance using advanced analytical tools such as the finite element method, as can be seen in the literature review. Consequently, this academic expansion has the potential to facilitate advancements in the design and application of polymer gears in various engineering and industrial contexts.

In their study, Vivek Karaveer and his co-authors [4] investigate tooth stress in intermeshing cylindrical gears to determine the maximum contact stress in the gear teeth. They use FEA to compare their findings with the theoretical values obtained from the Hertzian equation. The gears under examination are cylindrical in design and are made of materials such as steel and grey cast iron.

Rahul M. and D. P. Kamble [5] utilize the finite element method to analyze a set of cylindrical gears to determine their wear level. The Archard equation is used for wear assessment, and the numerical results are compared with experimental data. The material considered in this analysis is steel.

Rajeshkumar S. and Manoharan R. [6] present the results of FEA on cylindrical gears made from composite materials (glass fiber/epoxy). The Ansys 2023 parametric design language (APDL) was used to conduct the analysis. The study found that rotational speed is a significant parameter that influences the stress state of the gears. Three rotation speeds were employed in the simulations. The study shows that composite material is a superior choice compared to plastic and can even be a viable alternative to steel.

Bergant Z. et al. [7] present an engineering study on the fatigue and wear characteristics of carbon fiber-reinforced polymer (CFRP) gears. The study investigates the potential of CFRP gears as lightweight and high-performance alternatives to traditional gear materials. The wear test outcomes indicate the occurrence of adhesive wear and three-body abrasion wear mechanisms between the CFRP gears and their steel counterparts.

The aim of the paper [8] is to highlight the effect of the use of a composite body on the behaviour of hybrid metal-composite gears during engagement. The proposed model compares a hybrid pinion gear with a lightweight steel gear of the same mass to investigate the influence of the composite body on the stiffness of the gear. In the FEA simulations, the gear body is represented as a sequence of unidirectional layers of symmetrical composite, resulting in quasi-isotropic properties.

Recent years have seen the development of many polymers, which has led to extensive research into finding optimal materials for gears. Muratovic E et al. [9] explore the potential use of Polyvinylidene fluoride (PVDF) in gear applications and introduce an experimental setup to monitor the temperature generated during gear engagement. The study highlights the use of VDI 2736 to analyse tooth root stress and determine flank tooth pressure precisely. The investigation concludes that gears may fail due to elevated temperatures when Polyvinylidene fluoride is used. This tendency is more noticeable when there are substantial torsional moments, which increase the friction coefficient between the gear flanks.

The VDI 2736 German standard [10,11,12,13] highlights viable analytical solutions for polyamides (PA) and polyoxymethylene (POM), as emphasised in [9]. However, it lacks sufficient data for the use of other types of polymers in the context of cylindrical gears.

Further research and development in the field are necessary due to the limitations mentioned. Industries are exploring a broader range of polymer materials for gear manufacturing. The analytical solutions provided by Verein Deutscher Ingenieure 2736 (VDI 2736) for polyamides and polyoxymethylene serve as a foundation. However, there is a clear need to expand the standard to encompass a more diverse set of polymers commonly encountered in cylindrical gear applications. This expansion would contribute significantly to the comprehensive understanding and effective utilization of various polymer materials in gear design and manufacturing.

Hybrid couplings, which involve both plastic and steel components, are commonly used in engineering applications. Zhong B. et al. [14] conducted a comprehensive investigation into the degradation mechanisms of polyoxymethylene (POM) material by performing durability tests under diverse loading conditions. The study focuses on identifying the mechanism responsible for material degradation, specifically linked to contact fatigue. Contact fatigue is characterized by wear and damage resulting from repeated contact and loading cycles and is a critical factor affecting the performance and structural integrity of POM in mixed plastic-steel couplings.

Assessing noise levels in steel gear pairs is a common practice. This study explores the noise levels of the polymer couple POM/PA66 in detail [15] based on an experimental method. The researchers analysed the noise level generated by gears made from the combination of polyoxymethylene (POM) and polyamide 66 (PA66), comparing it with traditional steel gears. This approach significantly contributes to understanding the impact of noise levels in the use of POM/PA66 plastic gears, offering concrete and detailed data about their behaviour compared to traditional steel gears. The results obtained can be valuable for optimizing the design and selecting materials for specific applications where noise reduction is an important criterion.

Damijan Zorko’s study [16], examines the dynamics of cylindrical gears, with a focus on those made from plastic materials. The study highlights the importance of manufacturing quality in the operational performance of these gears. The investigation includes both experimental methodologies and numerical simulations, using the finite element method and the ANSYS 2023 program.

The study concludes that injection and cooling temperatures have a significant impact on the operational behaviour of plastic gears. Delrin 100 NC010 (POM-H) is identified as a suitable material for gears due to its robust fatigue resistance, minimal wear characteristics, and reduced coefficient of friction. The properties of the material contribute to its widespread use in gears, making it a preferred choice for applications where reliable performance is essential.

The lifespan of gears depends on the wear degradation process. Tunalioglu and Agca [17] conducted a comprehensive study on the wear behaviour of three materials integrated into cylindrical gears: polyethylene terephthalate (PETG), polylactic acid (PLA), and acrylonitrile butadiene styrene (ABS). Wear was assessed experimentally using a “Forschungsstelle für Zahnräder und Getriebebau” (FZG) type device. The study’s conclusion suggests that gears made from ABS and PLA have a shorter lifespan compared to the third material, even when subjected to identical demands.

Upon detailed analysis of wear behaviour, significant differences were found among the materials. The experimental findings were supported by analytical analysis, indicating that the reduced durability of gears made from ABS and PLA may be due to their more pronounced wear characteristics under similar operating conditions. The study presents a comprehensive perspective on the wear performance of PETG, PLA, and ABS materials in the context of cylindrical gears.

Despite these valuable studies, there remains a notable gap in the literature regarding polyamide 6 (PA6) gears fabricated through additive manufacturing techniques, especially when subjected to post-processing treatments such as heat annealing. The mechanical behavior, dimensional fidelity, and long-term stability of such components under realistic operational loads are not yet comprehensively understood.

This paper aims to bridge that gap by presenting a systematic investigation into PA6 gears produced via fused filament fabrication (FFF). It emphasizes:

- Process precision, including layer adhesion and geometric accuracy;
- Dimensional stability, both immediately post-printing and after thermal treatment;
- Realistic loading conditions, simulating operational stresses to assess performance and durability.

By addressing these aspects, the study contributes to the optimization of polymer gear manufacturing and expands the applicability of PA6 in functional mechanical systems.

## 2. Materials and Methods

### 2.1. Polyamide 6 (PA6)

The choice of materials for a technical application must be based on two principles:

- The materials selected must have physicochemical, mechanical and technological properties that meet the requirements of the application;
- The technical solutions for the intended use, in terms of their economic viability, in terms of development, semi-manufacturing and manufacturing costs.

The material selected for the research was the plastic PA6 (Filament POLYMAKER Fiberon PA6-CF20 Building 6&7&11, No.2, Hai Cheng Road, Chang Shu, Jiangsu Province, China). This material has a wide use in many fields, such as automotive, textile and packaging industries, due to its unique combination of excellent mechanical properties, chemical resistance, self-lubrication and barrier properties. Typically, polymers are processed by cooling the melt from the rapid melting temperature to room temperature, causing the polymer chains to struggle for equilibrium. A key role in these excellent properties is generally recognized to be played by hydrogen bonding interactions resulting from the amide groups between adjacent PA6 chains. Due to the presence of amide groups, PA6 absorbs significant amounts of water, up to about 10% by weight. The material used for the tests was PA6 without additives.

Specific properties of the PA6:

- Density: 1.15 g/cm^3^ (unfilled PA6 1.14);
- Thermal properties: T_g_ = 60 °C, T_m_ = 220 °C, HDT = 150 °C (unfilled PA6 70 °C).

### 2.2. Three-Dimensional Printing Process

The intricacies of the 3D printing process, specifically tailored for PA6, are elucidated in this section. The focus is on the rapid cooling phase and its implications for the final structural integrity of the printed samples.

**Figure 1:**
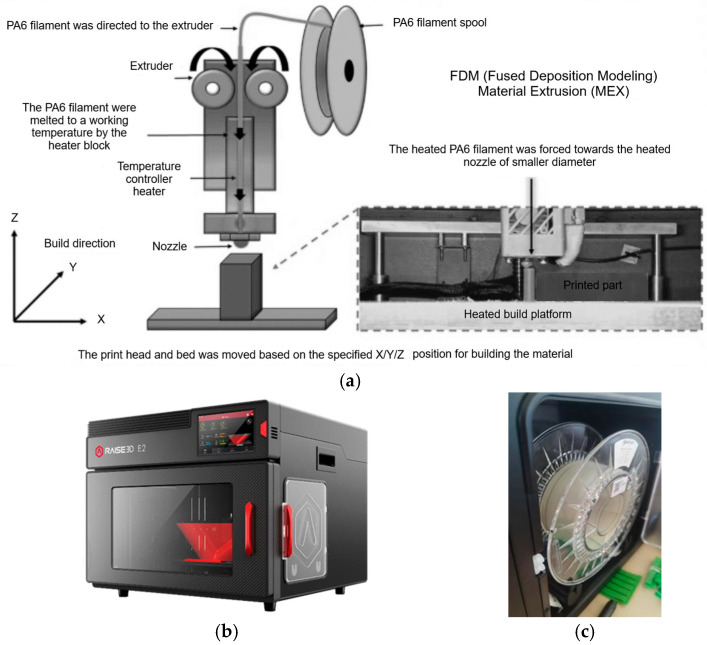
The Raise E2 3D printer from the Additive Technologies Laboratory of the Mechanical and Electrical Engineering Faculty: (**a**) process diagram for 3D printing a PA6 gear; (**b**) overview; (**c**) PA6 roll. (Source: authors based on the research course).

Process diagram of 3D-printed gear fabrication from PA6 (Figure 1a), highlighting printing parameters (layer height, nozzle temperature, bed temperature, infill density, orientation). This diagram emphasizes the critical stages influencing precision and dimensional stability. A process diagram for 3D printing a PA6 gear involves: 1. CAD Design; 2. Slicing (G-code generation); 3. Printing on an FDM 3D printer using specific parameters: nozzle temperature (e.g., 210–280 °C), bed temperature (e.g., 80–130 °C), layer height (e.g., 0.1–0.3 mm), infill density (e.g., 100%), and print orientation. This is followed by post-processing steps like support removal and quality inspection.

The Raise E2 3D printer − Raise E2 3D printers, Tenorweg 6, 3363LN, Sliedrecht, Netherlands (Figure 1b) from the Additive Technologies Laboratory of the Mechanical and Electrical Engineering Faculty was used for the printing of the samples, and a PA6 Neat roll (Figure 1c) was used for the printing of a large number of gears.

The main properties used for the production of the gears are those recommended by the manufacturer (Spectrum Industrial) and are shown in Table 1, [18]. The PA6 gears were manufactured using a fused filament fabrication (FFF) 3D printer. Printing was carried out with a nozzle temperature of 260 °C and a heated bed temperature of 90 °C. The printing speed was 50 mm/s, with a layer height of 0.2 mm and an interlayer bonding time of approximately 2 s. The infill density was set to 100% with rectilinear patterning to maximize structural integrity. After printing, the specimens were subjected to annealing treatments at different target temperatures ranging from 120 °C to 170 °C. The samples were heated at a controlled rate of 5 °C/min, held isothermally at the target temperature for 2 h, and subsequently cooled inside the oven (Snol of Lithuanian provenance (Snol, Umega Group, AB, SnolTherm Dpt., Narcűnai, LT-28104, Utena) at a rate of approximately 2–3 °C/min until reaching room temperature. These processing conditions were maintained consistently for all tested specimens to ensure comparability of results. The components were meticulously measured and weighed prior to testing. The components that exhibited the highest dimensional accuracy were selected for further analysis because this study investigates the impact of printing parameters on the precision of gears.

PA6 polymer is used when the temperature limit is reached or the hydrolytic stability of PA6 is insufficient. It provides good surface appearance and good adhesion, resulting in burst pressure resistance. An economic advantage is that printing cycles are fast.

PA6 is often used to replace metal in automotive parts where design flexibility, temperature and chemical resistance are critical due to its good processability. Lastly, they contribute to weight reduction, offering opportunities for CO_2_ reduction.

### 2.3. Test Methods

Some improvements in the mechanical properties can be observed when the bed temperature is increased from 60 °C to 85 °C; however, these changes do not lead to significant alterations in the overall material performance. In this study, the 3D-printed PA6 gears were subjected to annealing heat treatments within a temperature range of 60 °C to 200 °C, followed by air cooling. Based on mechanical testing, it was found that the most effective results were achieved within an optimal annealing range of 120 °C to 160 °C, where the mechanical properties of the material tend to stabilize and improve noticeably.

Heat treatments are used to enhance the mechanical properties of structures made of PA6 plastic material obtained through the 3D printing process, with a focus on improving tensile strength and yield strength. It should be noted that these treatments also cause minor changes in elastic properties, as explained in the preceding sections. This paragraph examines the impact of thermal treatments on the mechanical and elastic properties of PA6 materials and their effect on the operational behavior of cylindrical gears.

The study of operational behavior involves three primary methodologies: numerical, analytical, and experimental. Traditionally, the predominant focus in gear studies has been on metallic materials, particularly steel. The use of the finite element method (FEM) is prevalent in evaluating the performance and durability of gears, but there is a lack of research dedicated to analyzing gears made from plastic materials.

Gravimetric wear testing was conducted using a pin-on-disk tribometer (CSM Instruments, Freiburg in Breisgau, Germany) under dry sliding conditions at room temperature (20 ± 2 °C, relative humidity 45–50%). The PA6 samples, obtained from the printed gears, were tested against a hardened steel disk (Ra ≈ 0.2 µm). The applied normal load during the tests was approximately 900–1000 N, with corresponding maximum stresses in the range of 55–65 MPa and strains of 1.5–1.7%. The sliding speed was maintained at 0.2 m/s, and the tests were carried out for a total sliding distance of 1000 m, corresponding to a duration of about 85 min. After each test, the specimens were weighed on an analytical balance (type KERN ALJ, with ±0.1 mg accuracy) to determine the mass loss. Each condition was tested in triplicate, and average values were calculated.

Currently, there are numerous studies on the operational behavior of gears, using the finite element method to varying degrees. Most of these studies focus on assessing gears made of steel, with relatively fewer investigations dedicated to examining gears made of plastic materials. Although studies on steel gears provide valuable insights into mechanical performance, the growing use of plastic materials, especially in applications such as 3D printing, requires a more comprehensive examination of the operational behavior of plastic gears.

When applying the Finite Element Method (FEM) to analyse gears manufactured through 3D printing technology, it is important to have a nuanced understanding of the intricate relationships between several critical factors. These factors include the precision of FEM results, the definition of boundary conditions, the material characteristics intrinsic to the gears, and the accuracy of the geometric model.

The mechanical behavior and thermal properties of 3D-printed gears have a significant impact on their performance in different operational scenarios. It is essential to consider these material characteristics carefully to accurately replicate the mechanical response of the gears in a simulated environment.

Therefore, a robust Finite Element Analysis of 3D-printed gears requires a holistic approach. This requires precise calibration of boundary conditions, a comprehensive understanding of material properties, and the creation of a geometric model that accurately reflects the actual structure. This approach not only improves the accuracy of the simulation but also offers valuable insights into the performance and structural integrity of 3D-printed gears in practical applications.

## 3. Results and Discussion

### 3.1. Finite Element Analyses of 3D-Printed Gears

FEA simulations were conducted on cylindrical PA6 gears, applying torsional moments between 3000–7000 N∙mm at 100 rpm. Boundary conditions and contact models followed VDI 2736 guidelines. For computational efficiency, a sector containing five teeth was analysed.

The model assumed homogeneous isotropic PA6. However, in practice, 3D-printed PA6 is anisotropic due to weaker interlayer bonding. This limitation may overestimate predicted strength. A more accurate model would incorporate orthotropic properties or interlayer shear strength calibrated experimentally.

**Figure 2:**
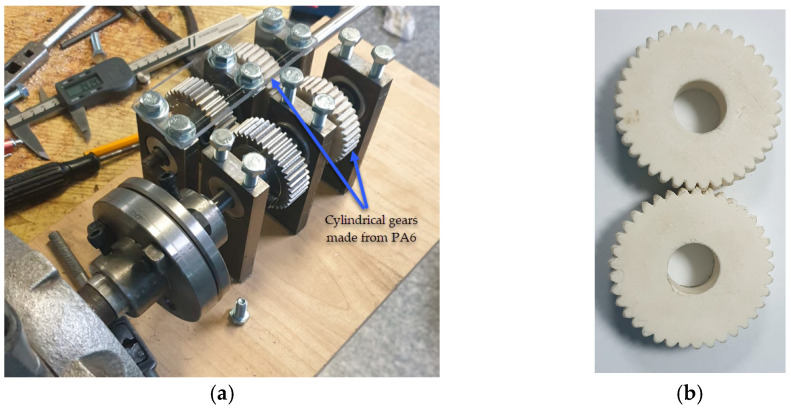
Experimental setup for tribological research; (**a**) experimental device for future tribological research; (**b**) PA6 gear. (Source: authors based on the research course).

Cylindrical gears made from plastic materials are often produced through the injection molding process, which is recommended for large-scale production runs. However, the gears being studied in this research were manufactured using the 3D printing process. Specifically, the material used was PA6, which was enhanced through a heat treatment process. These gearwheels have been included in an experimental setup for future tribological research (Figure 2).

**Figure 3:**
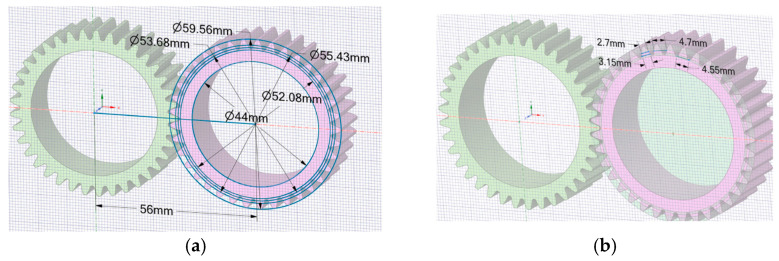
The geometric properties of the gears were numerically analysed: (**a**) reference diameters; (**b**) corresponding dimensions of the teeth. (Source: authors based on the case study).

Figure 3 and Table 2 present the geometrical characteristics of the gears that were analysed numerically (using FEA).

The decision to use 3D printing instead of injection molding for the cylindrical gears is significant as it deviates from the conventional manufacturing method. Injection molding, which is usually preferred for its mass production efficiency, is replaced by the AM process, highlighting the distinctive benefits of 3D printing technology.

The gears are made of PA6 material, which undergoes a heat treatment process to improve its properties. The decision to use heat treatment demonstrates a deliberate effort to customize the material properties to meet specific performance requirements.

**Figure 4:**
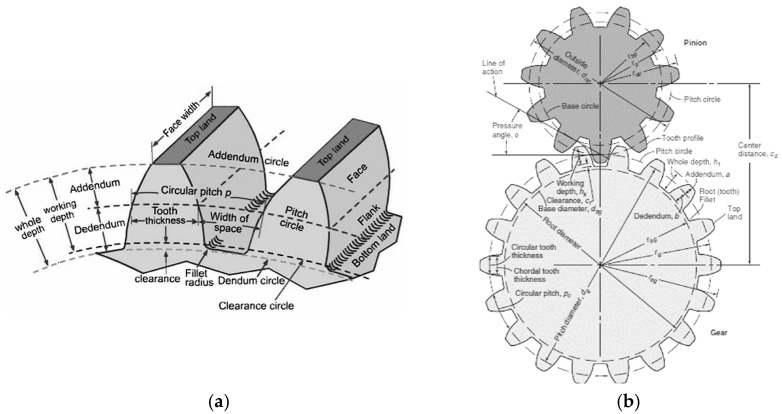
Gear wheel notations, adapted from [22]: (**a**) reference diameters; (**b**) meshing elements. (Source: authors based on the case study).

Figure 3 provides a comprehensive representation of the design of the 3D-printed gears, highlighting their geometric intricacies. Table 2 presents detailed specifications that offer a quantitative insight into various geometric parameters critical for the evaluation of the gears’ performance. This information complements the visual representation (Figure 4).

The characteristics for the material used to make the gears are detailed in Table 3, depending on the temperature values at which the heat treatment was conducted.

Table 3 contains data obtained on the basis of tests performed by the authors in their own laboratories, and the results are confirmed by the values reported in the literature, so that the sources cited in brackets certify the veracity of the results obtained.

**Table 3:** PA6 material properties involved in the FEA. (Source: authors based on the test results).

Parameters	Unit of Measure	Values
Young’s Modulus (in the scenario where the material is not heat-treated)	N/mm^2^	2100 ÷ 2800 [17,19]
Young’s Modulus (in the scenario where the material is heat-treated)	N/mm^2^	6000
Poisson’s Ratio	-	0.4 [17]
Ultimate Tensile Strength (in the scenario where the material is not heat-treated)	N/mm^2^	48 [17,19]
Yield Strength (in the scenario where the material is not heat-treated)	N/mm^2^	37 [17,19]
Ultimate Tensile Strength (in the scenario where the material is heat-treated)	N/mm^2^	67
Yield Strength (in the scenario where the material is heat-treated)	N/mm^2^	58
Density	kg/m^3^	1130 [10]
Wear factor	mm^3^/N∙m	(7.8 ÷ 9) · 10^−6^ [20,21]

A geometric model (3D) was created using Autodesk Inventor 2024^®^ (Educational Version) for the purpose of numerical analysis. The model was then transferred to Ansys SpaceClaim in the STL file format.

FEA on gear plastic wheel is often used to obtain detailed information about a system’s behaviour over time, using the transient analysis method [23].

The analysis framework used was the quasi-static model. To increase accuracy and reduce runtime, only a portion of the entire model was retained for analysis.

This portion includes five teeth for both the pinion and the driven gear, which will be engaged throughout the analysis. The appropriate boundary conditions were set and are presented below.

A three-dimensional model was used for numerical analysis, assuming homogeneous and isotropic materials. Torsional moments were applied to the pinion-wheel assembly with magnitudes of 3000 N∙mm, 5000 N∙mm, 6000 N∙mm, and 7000 N∙mm. The rotational speed considered was *v* = 100 rotations/min. The assumed coefficients of friction [1,24] between the pinion and the driven gear are 0.2, 0.28, 0.31, 0.35, and 0.28, respectively.

These assumptions form the basis for a comprehensive numerical analysis aimed at evaluating the mechanical behaviour of the system under different operating conditions. It is important to note that the assumption of homogeneity and isotropy in the materials simplifies the model, allowing for a more manageable mathematical representation. The applied torsional moments and coefficients of friction were thoroughly explored to understand the system’s response to various loading scenarios.

To achieve accurate numerical results, we paid meticulous attention to selecting input data and discretization parameters. The precision of the outcomes depended heavily on factors such as material characteristics, boundary conditions, and underlying assumptions. We also carefully considered finite element types and sizes, especially in regions with maximum stress.

### 3.2. Sensitivity Analyses

To determine the optimal size of finite elements for precise results, sensitivity analyses were conducted [25]. This involved systematically varying the dimensions of finite elements according to Table 3. During the discretization process, critical parameters such as the shape of elements, maximum aspect ratio, and maximum corner angle were thoroughly examined.

The analysis revealed that printing parameters such as layer height, nozzle temperature, and orientation influenced gear precision. Post-annealing, small but measurable contractions in reference and tip diameters were observed, with slight deviations in tooth flank alignment. These results confirm that both printing and heat treatment affect dimensional stability, which is crucial for gear meshing accuracy. The aim of this detailed investigation was to determine the impact of these parameters on result accuracy.

The sensitivity analyses involved creating multiple discretization models with varying finite element sizes, while maintaining consistent boundary conditions across all models. These conditions were rigorously applied to ensure a fair comparison. Subsequently, analyses were conducted on each model, and the results were meticulously compared with analytical solutions obtained through various methods such as the Lewis Bending Equation, the KISSsoft (release 2025) approach, and Classical beam theory.

**Figure 5:**
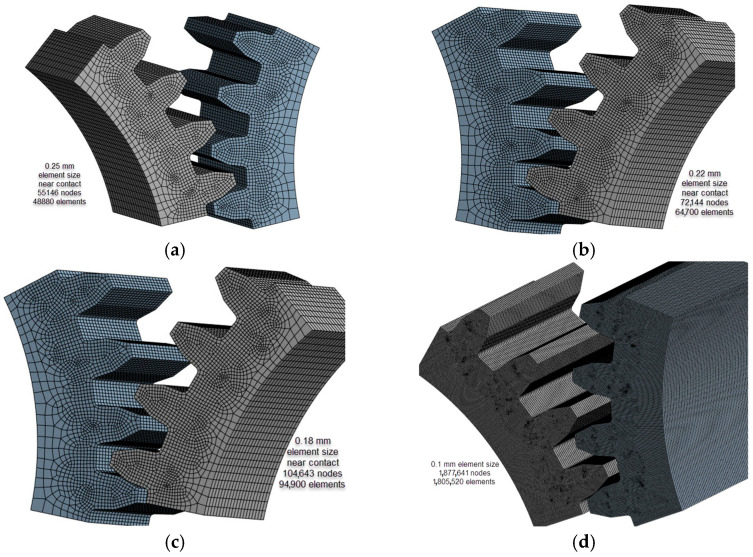
The models for discretization that correspond to the sensitivity analysis in element size expressed, mm: (**a**) 0.25; (**b**) 0.22; (**c**) 0.18; (**d**) 0.1. (Source: authors based on the results of the sensitivity analysis).

**Figure 6:**
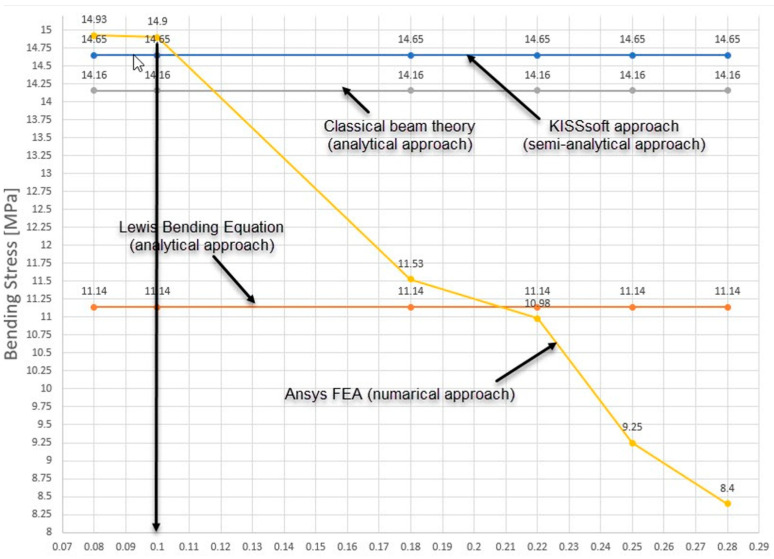
The results of the sensitivity analysis. (Source: authors based on the sensitivity analysis).

As shown in Figure 5 and Figure 6 and Table 4, the discretization model using finite elements with element dimensions of 0.1 mm in the regions corresponding to the contact between the pinion and the driven gear closely aligns with the analytically obtained results. The bending stress is prominent at the base of the engaged tooth, and analytical calculations exhibit good agreement, especially in the case of the KISSsoft approach and Classical beam theory (only 3.34%). Regarding the differences in bending stress between FEA and KISSsoft, the difference is 1.7% (and 5.22% between FEA and Classical beam theory).

This indicates that the discretization model with 0.1 mm finite element dimensions in the contact regions between the pinion and the driven gear (Figure 5d) yields results that closely align with those obtained analytically. Regarding bending stress, there is notable agreement, particularly when comparing the KISSsoft approach and Classical beam theory, with minimal discrepancies between finite element analysis (FEA) and analytical methods.

In Table 4 are presented the comparative stress results between the approached methods (FEA-analytical) for the bending test.

**Figure 7:**
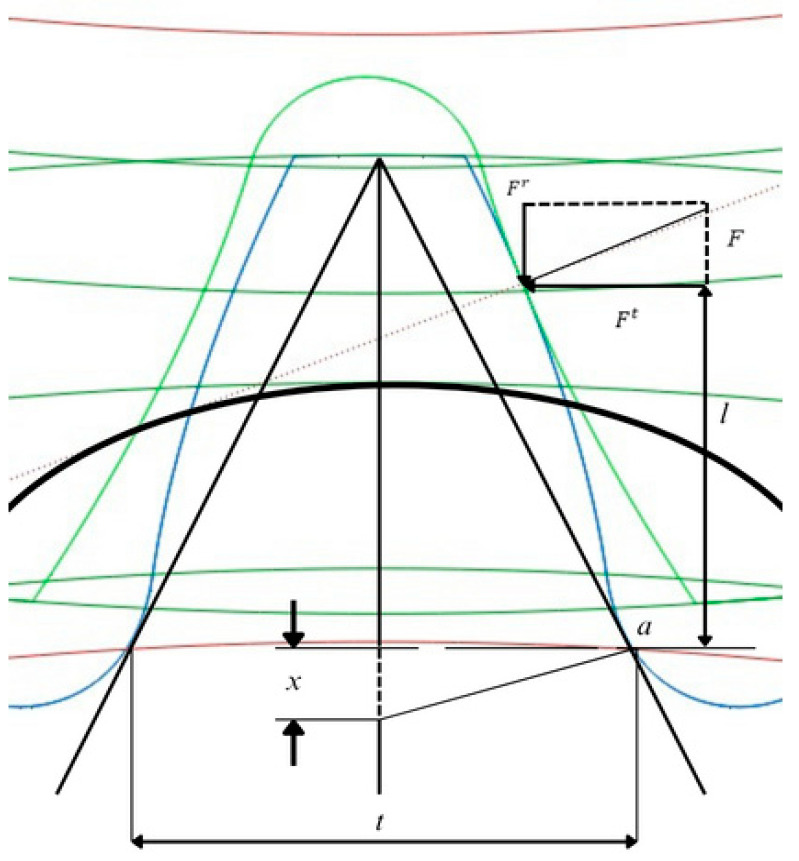
The calculation scheme associated with classical beam theory. (Source: authors based on the analysis results).

The maximum differences observed, especially in the comparison between FEA and Lewis Bending Equation (Figure 7), are within a reasonable margin of 25%, highlighting the reliability of the finite element analysis results.

The significance of the notations used in the evaluation of bending stress within Table 4 is: σ—bending stress, MPa; *F^t^*—pinion tooth force, N; *t*—tooth thickness at base, mm; *I*—inertia moment of the cross-sectional area of the tooth, mm^4^; *S_cf_*—Stress concentration factor at tooth root, *S_cf_* = 1.25, [11]; *l*—contact height, mm; *w*—face width, mm; *m*—wheel teeth module; *Y*—The Lewis factor depends on the number of teeth and the pressure angle. For the studied gear, [21], it is reported as 0.36.

The study ensured the reliability and precision of the simulation outcomes by benchmarking the numerical results against established analytical methods. This approach enhanced confidence in the accuracy of the results and underscored the thoroughness of the methodology employed in the analysis.

In terms of load, the sensitivity analysis models (Figure 5) were subjected to a torsional moment of 3000 N∙mm. The material used was untreated PA6, which is described in Table 3, and the dimensions were in accordance with Table 2.

The pinion and driven gear components were modelled in the numerical model using SOLID185 finite element types, providing a three-dimensional representation. Tooth contact regions were modelled using CONTA174 and TARGE170 elements. CONTA174 is a contact element used to model surface-to-surface contact, while TARGE170 is used for target elements in contact analysis. The MPC184 element type is used to define and model boundary conditions. The MPC184 element, or Multipoint Constraint element, is useful for connecting multiple degrees of freedom at different points, allowing for the accurate representation of complex boundary conditions.

**Figure 8:**
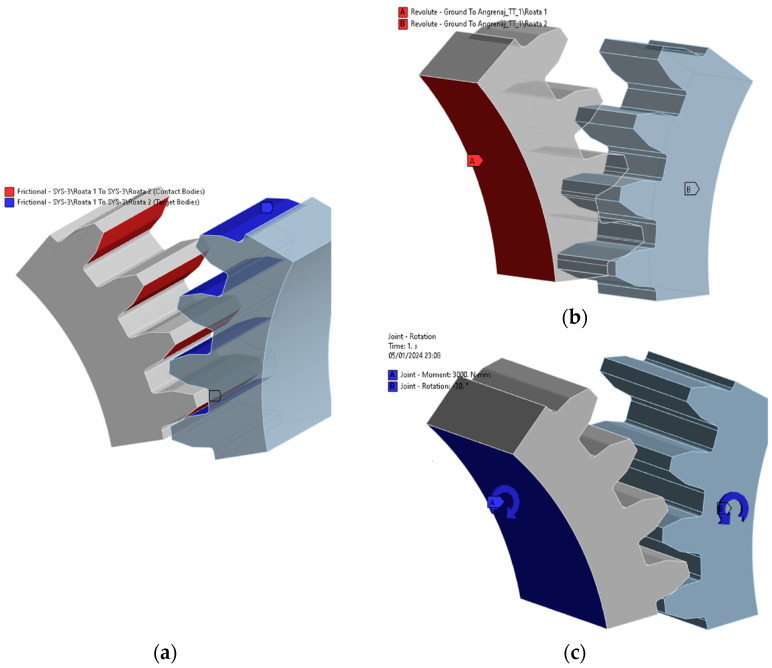
Boundary conditions: (**a**) frictional surfaces; (**b**) revolute surfaces; (**c**) joint moment and rotation. (Source: authors based on simulation results).

The boundary conditions (Figure 8) adopted are of the ‘Joint-Revolute’ type, which represent rotational couplings defined at the shafts of the two gears. These connections enable the rotation of the two gears around their respective axes. The applied rotational moment, introduced on the pinion, is a ‘Joint Moment’ load that is constant with a value of 3 N∙m.

On the tooth flanks that are or will engage, the contact is of the ‘Friction’ type, with a friction coefficient value of μ = 0.4 [1,24] (only for sensitivity analysis).

A ‘friction contact’ model is employed for the tooth-to-tooth contact on the engaging flanks. The given simulation uses a friction coefficient of μ = 0.4 [1,24] to represent the interaction between the contacting surfaces. It is important to note that this value is only used for the sensitivity analysis. The boundary conditions and contact models together create a realistic representation of the mechanical interactions within the gear system, capturing both the rotational constraints at the shafts and the frictional effects at the tooth contact points. This comprehensive approach ensures a detailed and accurate simulation of the gear behaviour under the specified loading conditions.

### 3.3. Effect of Heat Treatments on Bending Stress and Flank Contact Pressure

The bending stress values and contact pressure of cylindrical gear teeth are influenced by the geometric configuration of the gear system, the nature of the loads, and the elastic and mechanical characteristics of the materials involved. The evaluation method for gears made of plastic materials (PA6) is specified in VDI 2736, introduced in 2014. VDI 2736-2 [11] outlines the procedure for evaluating bending stress in cylindrical gears based on the equations from Table 5 [11,19,26].

While the baseline simulations assumed constant torque and rotational speed, real-world gears encounter fluctuating loads, shocks, and temperature variations. Future extensions should include thermo-mechanical coupling and dynamic load cycles to evaluate gear performance under conditions such as automotive transmissions.

Like the assessment of bending stress, which is obtained at the base of the teeth in contact, contact pressure can be evaluated based on VDI 2736-2.

The equation used for this evaluation is [11,16]:

(1) $$\sigma_{H}=Z_{E}\cdot Z_{H}\cdot Z_{\varepsilon }\cdot Z_{\beta \cdot }\sqrt{\frac{F_{t}\cdot K_{H}}{b_{w}\cdot d_{1}}\cdot \frac{u+1}{u}}\leq \sigma_{HP},$$

where $\sigma_{H}$ represent the flank pressure at the contact point in MPa.

When evaluating contact pressure between gears, it is important to consider the elastic material properties. It is important to maintain a balanced and objective evaluation of these properties. The elasticity factor, $Z_{E},$ considers material characteristics such as the longitudinal modulus of elasticity and Poisson’s ratio. These properties determine how the material responds to the stresses during gear engagement, which affects the overall contact pressure.

The zone factor, $Z_{H}$, draws attention to the geometric aspects of cylindrical gears in contact. It includes features related to the design and configuration of the gears, providing insights into how their geometry affects the distribution of forces and stresses in the contact area. This factor is important for tailoring gear designs to achieve optimal performance and longevity. Moving beyond material and geometric considerations, the contact ratio factor, $Z_{\varepsilon }$, delves into the effective contact length between gear flanks. This parameter is particularly significant as it accounts for the duration and extent of contact between the teeth during rotation. Understanding the effective contact length is important for predicting wear patterns, fatigue, and optimizing gear designs for durability.

Finally, the $Z_{\beta \cdot }$ factor represents the spiral angle and captures the helix angle of the gears. It usually has a value of one, indicating straight-toothed gears. However, its inclusion highlights the significance of considering gear helix in specific applications. The spiral angle affects tooth engagement dynamics, which impacts factors such as load distribution and noise generation. Evaluating contact pressure requires considering elasticity, geometry, effective contact length, and helix angle factors. These parameters collectively contribute to understanding the interplay between material properties and gear design, enabling engineers to make informed decisions for optimized gear performance.

The permissible contact pressure $\sigma_{Fp}$ [11,16] is determined by the relationship between the fatigue strength of the material, $\;\sigma_{H_{limN}}$, in contact and the surface roughness, through the $Z_{R}$ factor. $Z_{R}$ is generally equal to unity, and the safety factor coefficient $S_{H_{min}}$ is set at 1.4, which determines the permissible pressure.

(2) $$\sigma_{\mathrm{Fp}}=\frac{\;\sigma_{H_{limN}}}{S_{H_{min}}}\cdot Z_{R}$$

Based on the analytical relationships presented earlier, an evaluation of bending stress and contact pressure was carried out using the KISSsoft program, which implements the expressions specified in the VDI 2736-2 standard, as well as through numerical analyses (FEA-Ansys 2023), [28]. Simulations were conducted to draw conclusions on the influence of heat treatments on PA6. The study compared untreated PA6 to PA6 that underwent heat treatment at 160 °C annealing temperature. Table 6 and Table 3 present the mechanical and elastic characteristics, respectively, while Table 2 provides the geometric details as previously mentioned.

To compare and validate the simulation results, Table 6 and Table 7 summarize the outcomes. The finite element model in Figure 5d is used, with boundary conditions previously shown in Figure 8.

**Figure 9:**
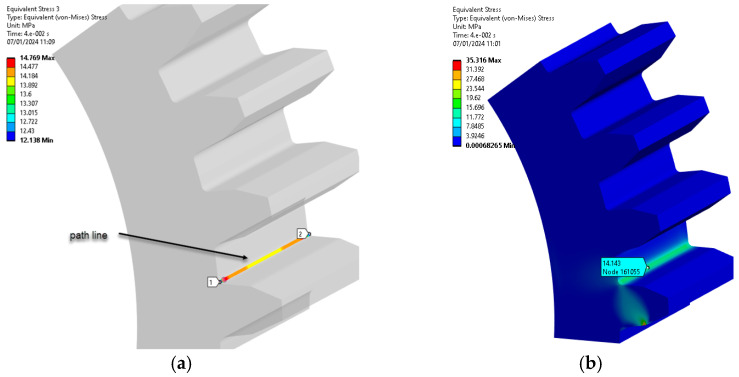
Path line and bending stress (PA6 untreated): (**a**) stress on path line; (**b**) bending stress distribution, MPa. (Source: authors based on the bending stress simulation results).

The ‘path’ command in Ansys 2023 Workbench highlights bending stresses by selecting nodes within the region of maximum load on the tooth, as seen in Figure 9. The meaning of the colors in all figures showing the results of the ANSYS simulation can be found on the left side of each figure. The colors represent the intensity of the mechanical stresses, with red representing the maximum stress.

**Figure 10:**
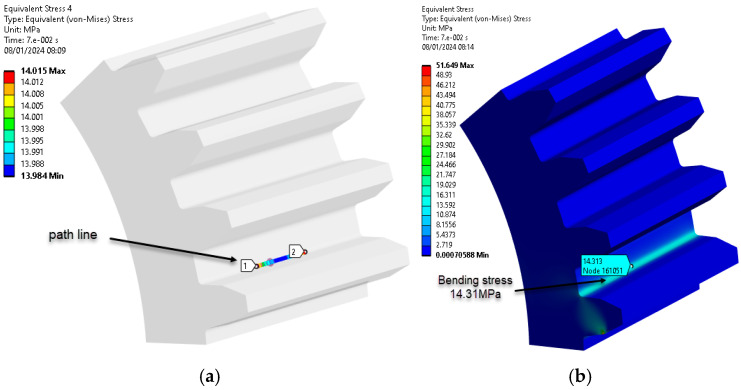
Path line and bending stress (PA6, 160 °C annealing temperature): (**a**) stress on path line; (**b**) bending stress distribution, MPa. (Source: authors based on the bending stress simulation results).

Figure 9 and Figure 10 show the path line and bending stress.

**Figure 11:**
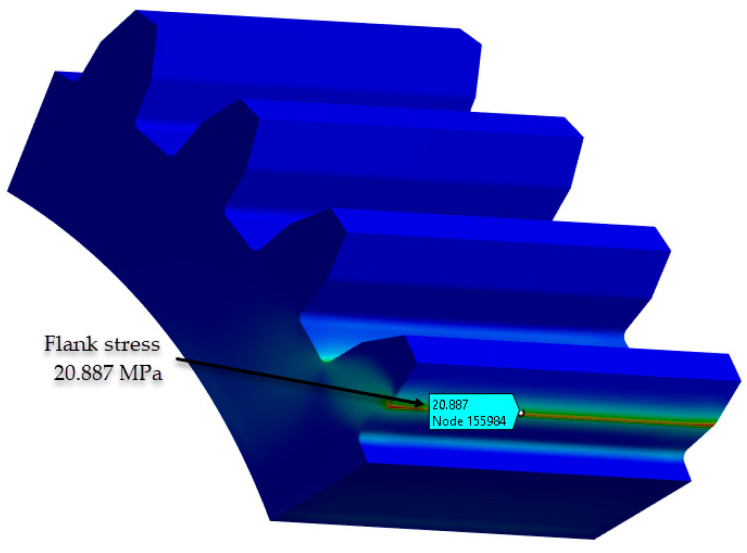
Stresses in the contact zone (PA6 untreated). (Source: authors based on the bending stress simulation results).

The procedure for identifying and validating the flank stress calculation was applied in the same way. The stresses in the contact zone between the flanks can be found in Figure 11.

The ‘path’ command in Ansys 2023 Workbench simplifies the selection of nodes that are critical for assessing the maximum stresses in the tooth, enabling a detailed analysis of the variation in equivalent stress.

The data presented in Table 6 and Table 7 show a commendable agreement between the results obtained analytically and those obtained by numerical means, in particular FEA. As expected, the largest discrepancy of 9% is found in the flank stress calculation. This modest discrepancy is rationalised by the influence of coefficients within the analytical relationships, as prescribed by VDI 2736-2.

The sensitivity of these coefficients is evident, as their selection has a relatively small but noticeable effect on the resulting values. This comparative analysis underlines the robustness of both analytical and numerical methods, while emphasising the importance of careful selection of coefficients in analytical formulations to ensure accurate predictions in engineering applications.

### 3.4. Effect of Heat Treatments on Wear and Service Life

The primary method for measuring wear involves testing a sample of the material with a rotating ring, using a pin-on-disk tribometer. This technique involves evaluating a material’s wear resistance by subjecting it to controlled friction and abrasion processes. A specimen, often in the form of a pin, is pressed against a rotating disk, simulating dynamic contacts resembling real-world operational conditions. Through this apparatus, wear processes can be simulated, allowing for a detailed analysis of the material’s response to both frictional forces and abrasive wear. This method provides valuable insights into the material’s durability and performance under conditions that mimic practical usage scenarios.

Annealing between 120–160 °C improved the value of Young’s modulus and tensile strength, leading to reduced tooth root stress and extended service life. However, excessive treatment (e.g., 160 °C) increased stiffness but also accelerated wear due to higher flank pressures. The balance between stiffness and wear resistance is therefore critical.

The analysis focuses on three core parameters: the normal load applied to the specimen, the rotational speed of the disk, and the sliding distance travelled by the material against the rotating ring. These factors collectively influence the wear rate, a critical metric that indicates material degradation over time. The Archard wear Equation (3) provides a quantitative framework for this assessment, factoring in the volume of material loss, the normal load, and the sliding distance.

(3) $$W=\frac{K}{H}\cdot F_{n}\cdot l,$$

where *W* in mm^3^ represents wear volume; *K* in mm^3^/N∙m is the wear factor (depends on the combination of materials); *H* is the hardness of the gear surface materials; *F_n_* in N is the normal force; and *l* in mm is the sliding distance [29].

Abrasive wear is a critical consideration, especially in scenarios involving dry running conditions for cylindrical gears. The absence of lubrication exacerbates the challenges associated with abrasive wear in gear systems. Solid contaminants such as dust and particulate matter have a more direct and pronounced impact on the contacting surfaces of the gear teeth in the absence of a lubricating film. The abrasive particles act as cutting agents, leading to accelerated material removal and surface deterioration. This phenomenon is exacerbated by the absence of a lubricating barrier that could otherwise reduce friction and wear.

VDI 2736-2 highlights local linear wear in cylindrical gears made from plastic materials using the following expression, [11,30]:

(4) $$W_{l}=\frac{F_{n,l}}{b}\cdot k_{w}\cdot N\cdot v$$

The symbol $F_{n,l}$ denotes the local normal force in N; *N* stands for the quantity of loading cycles; *b* is the face width in mm; $k_{w}$ is the wear coefficient measured in mm^3^/N∙m; and v, mm is slippage.

An interesting algorithm for wear assessment is presented in [3,31,32]. The calculation formula, which considers the contact pressure between flanks and sliding distance is highlighted using the finite element method. The wear assessment formula is defined by the relationship [3,31,32]:

(5) h=k·s·P,

where [30] *h*, mm represents wear depth; k is wear rate in mm^3^/N∙m; *P*, MPa is the contact pressure; and *s*, mm is the sliding distance.

The relationship between wear and wear depth is dependent on the number of loading cycles, as shown by the formula:

(6) $$h\left(N\right)=k\cdot s\left(N\right)\cdot P\left(N\right),$$

where *N* represents the cycle number. This indicates that in the context of FEA, an iterative calculation is necessary to accurately evaluate wear.

The procedure involves establishing the model associated with the cylindrical gear for assessment, conducting FEA analysis, and determining the initial wear depth, denoted as h (initial). The initial iteration begins by adjusting the original model based on the wear depth and subsequently re-conducting the FEA analysis to determine the updated wear depth. The iterative process continues guided by the principle that modifications are made to the model after each cycle until the predetermined number of cycles is achieved [27]

The connection between the iterative process and wear is significant. As wear progressively affects the geometry and material properties of the system, each iteration reflects an evolving state of the gear mechanism. This iterative cycle enables a dynamic adjustment of the model, considering the changing conditions caused by wear.

The iterative nature of the process is essential due to the reciprocal relationship between wear and the system’s response to mechanical loading. The model is iteratively adjusted to account for changes, ensuring that subsequent analyses capture the evolving nature of wear.

To evaluate the impact of heat treatments on the wear behaviour of cylindrical gears with teeth made of PA6 materials, we analysed the geometric model shown in Figure 2. The model has mechanical characteristics as per Table 3, using analytical principles (VDI 2736-2, KISSsoft – KISSsoft AG, Rosengartenstrasse 4, 8608 Bubikon, Switzerland).

Table 8 presents the results of applying the calculation expressions, specifically Equation (4), for an initial non-iterative calculation, and Table 9 presents the results of applying the calculation expressions, specifically Equation (4), for an iterative calculation.

The mechanical characteristics of the materials from Table 8, respectively Table 9, are provided in Table 3. The considered wear coefficient in the analysis was *w* = 9 mm^3^/N∙m [20,21].

**Figure 12:**
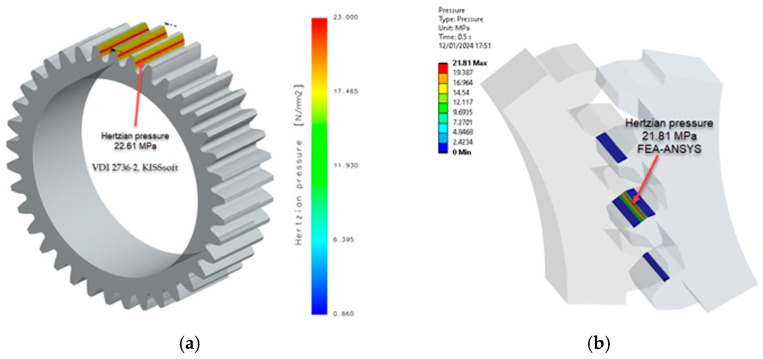
Contact (hertzian) pressure PA6, non-treated: (**a**) non-iterative calculation VDI 2736-2, KISSsoft; (**b**) FEA-Ansys 2023. (Source: authors based on the simulation results).

**Figure 13:**
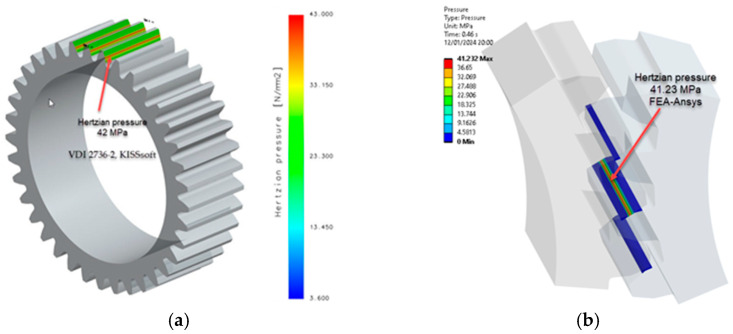
Contact (hertzian) pressure PA6, head-treated (E = 5000 MPa): (**a**) non-iterative calculation VDI 2736-2, KISSsoft; (**b**) FEA-Ansys 2023. (Source: authors based on the simulation results).

**Figure 14:**
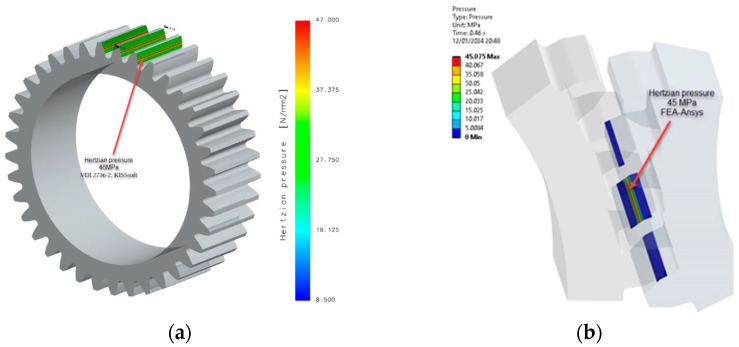
Contact (hertzian) pressure PA6, head-treated (*E* = 6000 MPa): (**a**) non-iterative calculation VDI 2736-2, KISSsoft; (**b**) FEA-Ansys 2023. (Source: authors based on the simulation results).

Wear depends on several factors, including contact pressure, which plays an important role. The contact pressure (hertzian pressure) values from Table 8 were compared with those obtained through numerical simulation (FEA-Ansys 2023), resulting in a highly favourable correlation, as shown in Figure 12, Figure 13 and Figure 14. This observation highlights the robustness of the simulation results and their alignment with the analytical contact pressure values. This correlation improves the reliability and validity of the data obtained, providing a strong foundation for further analysis and conclusions.

The results presented in Table 8 demonstrate that the assessment of flank wear through non-iterative calculation is not affected by the nature of the PA6 material, indicating independence from heat treatment. Additionally, the other parameters, except for the Hertzian pressure values, which increase with the longitudinal elastic modulus, fall within a relatively narrow range. However, when assessing wear, iterative calculations are necessary. Therefore, data obtained through non-iterative methods may not be as precise and should be considered with caution.

**Figure 15:**
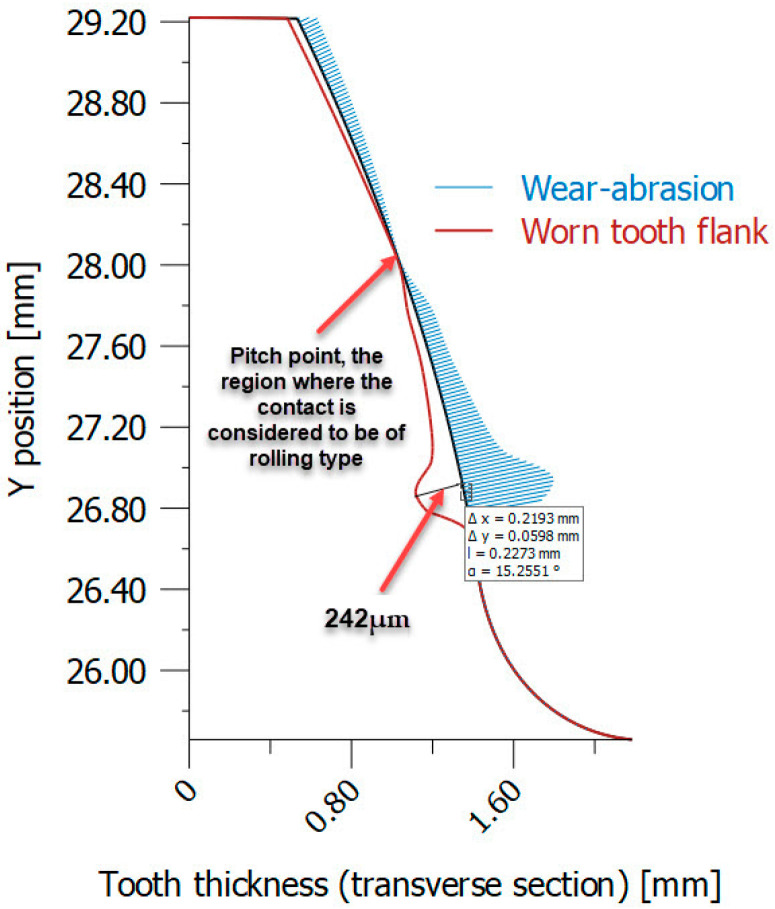
Worn tooth flank distribution in case of PA6 (heat-treated) using non-iterative simulation (VDI 2736-2, KISSsoft). (Source: authors based on the simulation results).

The use of non-iterative simulation results for wear assessment is not preferred due to the lack of objectivity. This is further highlighted by the examination of the wear distribution across the profiles of teeth fabricated from PA6 plastic material, as shown in Figure 15. The distribution was derived for PA6 material that underwent heat treatment with a longitudinal elastic modulus (*E*) of 6000 MPa. Figure 15 shows a significant absence of wear at the contact point between meshing teeth. The phenomenon described can be attributed to the simplification inherent in non-iterative simulations. In these simulations, contact is assumed to involve rolling rather than the more realistic sliding behaviour.

The implications of this observation are significant. This neglects the complexities introduced by sliding, which is a more accurate representation of real-world scenarios. Non-iterative simulations may not fully capture the nuances of wear behaviour at tooth contact points. This is especially relevant when dealing with plastic materials like PA6, where wear patterns can be influenced by factors such as material properties, heat treatment, and contact conditions.

The comparison between non-iterative and iterative wear models illustrates that while the non-iterative approach assumes constant contact conditions, the iterative model continuously updates contact geometry and pressures, providing a more accurate prediction of progressive wear with experimentally validated input parameters. In this study, a comparison is drawn between two models of wear. It is highlighted that the non-iterative approach, under the assumption of constant contact conditions, provides a simplified estimate of wear. In contrast, the iterative model, which updates contact geometry and pressure distributions at each step, allows for a more accurate prediction of progressive wear under conditions that have been experimentally validated.

The difference between simulated and real-world wear distributions highlights the need for iterative approaches to capture the complexities of contact behaviour and wear. This ensures a more accurate representation of the physical phenomena involved. Therefore, it is important to exercise caution when interpreting non-iterative simulation results for wear assessment, particularly in contexts where a high degree of precision is required for reliable engineering analyses and design considerations.

When conducting iterative analyses using the same materials and solicitation conditions, the results presented in Table 9 reveal a clear trend. This highlights the discernible influence of the elastic and mechanical characteristics obtained through heat treatments on the wear process. The parameters of local linear wear (µm), wear volume per tooth (mm^3^), and wear mass per gear (g) increase notably with the longitudinal modulus of elasticity. This highlights the significant influence of the longitudinal modulus of elasticity on wear behaviour, demonstrating a direct correlation between the mechanical properties obtained through heat treatments and the resulting wear characteristics. The iterative nature of the analyses provides a strong basis for asserting the significance of these thermal treatments in influencing wear dynamics. This emphasizes the complex interplay between material characteristics and wear outcomes. The validation of the numerical results for iterative simulations of material loss due to wear is detailed in Table 10. This validation involves comparing the numerical results with experimental data obtained from the set-up shown in Figure 2. Notably, the maximum difference in percentage material loss is 4.17% for untreated PA6. In addition, the material loss due to wear tends to increase as the Young’s modulus increases. This observation is supported by the experimental results. This indicates a clear relationship between the mechanical properties of the material and its wear behaviour, suggesting that materials with higher stiffness may experience greater wear under similar conditions. Further research into the causes of this increased wear and the potential effects of different material treatments would provide valuable insights for future research and practical applications in engineering, particularly in the selection of materials for wear-sensitive applications.

**Figure 16:**
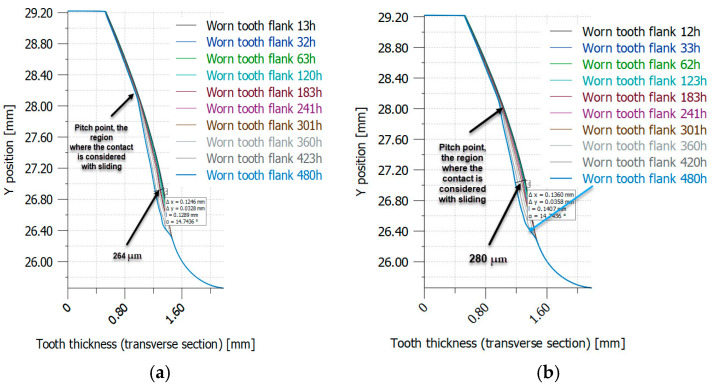
Worn tooth flank distribution in case of PA6 (heat-treated) using iterative simulation (VDI 2736-2, KISSsoft): (**a**) PA6 not heat-treated (*E* = 2100 MPa); (**b**) PA6 heat-treated (*E* = 5000 MPa). (Source: authors based on the simulation results).

**Figure 17:**
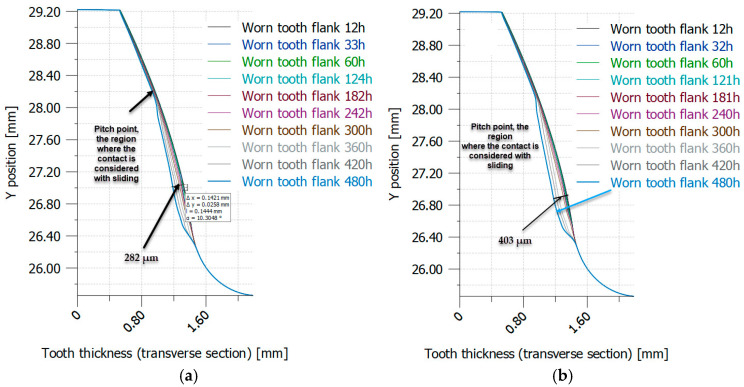
Worn tooth flank distribution in case of PA6 (heat-treated) using iterative simulation (VDI 2736-2, KISSsoft): (**a**) PA6 heat-treated (*E* = 6000 MPa); (**b**) PA6 heat-treated (*E* = 8000 MPa). (Source: authors based on the simulation results).

Figure 16 and Figure 17 show the distribution of worn tooth flanks in heat-treated PA6, as determined through iterative simulation (VDI 2736-2, KISSsoft).

## 4. Discussions

### 4.1. Discussions About Case Study

Cylindrical gears are subjected to a variety of stresses, the performance of which is contingent upon the material and design employed. Two methods for enhancing the mechanical properties of gears are heat treatments and the incorporation of fibers. The application of heat treatment is an effective method for modifying the mechanical properties of polymers, thereby expanding their range of potential applications. This process entails the controlled heating or cooling of the material under specific conditions, which results in structural changes at the molecular level. Heat treatment can induce alterations in crystallinity, molecular orientation, and chain mobility, which significantly influence the properties of polymers. By subjecting the material to heat treatment or incorporating fibers, the mechanical characteristics of gears, regardless of whether they are made of metal or plastic, can be enhanced, thereby improving their performance and durability.

Heat treatment is employed to enhance the mechanical properties of polymers, including their strength, stiffness, and resilience. By regulating the thermal parameters throughout the treatment process, the configuration of polymer chains can be modified, resulting in a more ordered and structured material. This increase in crystallinity contributes to enhanced strength and stiffness. Furthermore, controlled cooling or annealing can serve to reduce internal stresses within the polymer structure, thereby enhancing its toughness and resilience. The application of heat treatment to polymers results in an enhanced resistance to deformation, wear and fatigue, rendering them more durable and reliable, suitable for use in demanding applications. Furthermore, the incorporation of fibers during the heat treatment process can lead to an additional improvement in strength, stiffness and resistance to deformation. The objective of combining heat treatment with fiber reinforcement is to achieve a comprehensive enhancement in the mechanical characteristics of gears. In conclusion, heat-treated polymers offer a broader range of applications and are stronger and more reliable.

The optimisation of gear design and the assurance of reliable operation necessitate an understanding of the relationship between material properties and gear performance. One method of modifying the mechanical properties of cylindrical gears is through the application of heat treatments and fiber reinforcement. By examining the effects of heat treatments on polyamide PA6, a material renowned for its strength and versatility, researchers are able to elucidate the changes induced by thermal processes.

To investigate the behaviour of PA6 gears during operation, a physical model was created using 3D printing technology. This model was then subjected to two types of simulations: numerical simulations using FEA with Ansys 2023 software, and analytical simulations based on the principles outlined in VDI 2736-2 code using KISSsoft software. The insights gained from these simulations provide valuable data on the operational challenges that cylindrical gears face over their lifetime.

The authors conducted a quasi-static analysis utilising rotational speed and torque parameters to investigate the effects of heat treatments on the mechanical and elastic properties of PA6 materials. The authors employed experimental data obtained from mechanical tests as input parameters for their numerical analyses. The results of the sensitivity analysis, as illustrated in Figure 3, were employed to calibrate the numerical model. By incorporating directly obtained experimental data, this study is distinguished from existing literature, with the objective of enhancing our comprehension of the impact of heat treatments on the behaviour of cylindrical gears during operation. The combination of numerical analysis and analytical evaluations enhances the reliability and robustness of the findings, facilitating cross-validation and a more nuanced interpretation. Moreover, the incorporation of the VDI 2736-2 code in the analytical studies aligns the research with industry standards, thereby increasing the practical applicability of the results.

This study is grounded in scientific rigor and practical relevance, adhering to established codes and standards. The research offers valuable insights into the performance and durability of heat-treated PA6 gears, indicating that fatigue and material wear are the primary sources of stress. The study highlights the significance of these factors in determining gear longevity and reliability. Additionally, it acknowledges flank contact pressure as an important stress factor, enhancing comprehension of the operational challenges encountered by these gears. The findings indicate that bending stress at the base of the teeth exerts a relatively minor influence on the overall mechanical behaviour of the gears. This stress ranking facilitates the prioritisation of design considerations and maintenance strategies to enhance the longevity and efficiency of heat-treated PA6 gears in real-world applications.

The objective of the heat treatments was to enhance the tensile strength, yield strength, and fatigue characteristics of the material by increasing the number of cycles to failure. The tables presented in the study clearly demonstrated the positive effects of the heat treatments. Moreover, the study revealed that these heat treatments also led to a considerable enhancement in the longitudinal modulus of elasticity (E). While the modulus of elasticity of untreated PA6 is typically around 2100 MPa, the applied treatments increased it to 6000 MPa. This increase in modulus of elasticity is found to have a direct correlation with high contact pressures. The study establishes a clear link between heat treatments, modulus of elasticity, and contact pressure, highlighting the importance of these factors in determining the performance of PA6 materials in gear applications.

The stress values presented in Table 6 and Table 7 were determined through the application of analytical methods to PA6 gears under specific loading conditions, including a torsional moment of 3000 N∙mm, a rotational speed of 100 rpm, and a friction coefficient of 0.4. The aforementioned loading conditions remained consistent regardless of the type of heat treatment applied. The results obtained from the different heat treatments showed variations in mechanical and fatigue characteristics. However, the impact of these variations on the evaluation of allowable tooth root stress and allowable contact stress was found to be significant. Table 6 demonstrated that the modulus of elasticity (E) had minimal influence on the tooth root stress, as supported by finite element analysis. Conversely, Table 7 indicated that the modulus of elasticity significantly affected the contact stress at the operating pitch circle and nominal contact stress.

The present study examines the relationship between material properties and the distribution of stress in gears. The modulus of elasticity was found to exert a greater influence on contact stress and nominal contact stress, while tooth root stress was less affected. This understanding is of great importance for the accurate prediction of the mechanical behaviour of gears under different heat treatment conditions. Additionally, the fatigue properties of heat-treated PA6 materials were identified as exerting a considerable influence on gear service life and wear behaviour. It is recommended that future research should focus on obtaining fatigue curves for specific heat treatment conditions. Furthermore, the study found that samples that had not undergone any treatment and samples that had been treated at 120 and 140 °C exhibited similar gear life, despite variations in contact stress. However, the sample that had been treated at 160 °C broke this pattern with a significantly reduced life of only 1024 h, which was attributed to the absence of wear between the contacting flanks.

The wear of gear wheels, as outlined in relation (4), is markedly influenced by contact pressure and the number of loading cycles, both of which are affected by the fatigue characteristics resulting from heat treatments. The study elucidates the intricate interrelationship between material properties, contact pressures, and fatigue characteristics, demonstrating that gear wheels with disparate treatments can exhibit comparable longevity. This indicates that while variations in the modulus of elasticity can influence contact stress, they may not be the principal determinant of gear wheel durability. Conversely, it can be seen that the overall durability and lifespan of the gears are more substantially influenced by wear and associated mechanisms, which are themselves influenced by contact pressure and loading cycles.

In order to gain a deeper understanding of gear performance and durability, it is essential to consider wear-related aspects, such as contact pressure and loading cycles. This approach can provide valuable insights into potential optimisations for gear design and material treatments. The research indicates the necessity for a focus on wear mechanisms in order to enhance the performance of gears. Furthermore, Table 10 outlines the minimum fatigue strength requirements under pulsating stress and rolling contact fatigue strength for PA6, which must be achieved through heat treatments. These values emphasise the importance of selecting appropriate heat treatment processes in order to ensure that the material can withstand the stresses encountered in gear applications, which ultimately leads to improved gear durability and performance.

Fatigue strength under pulsating stress, also known as nominal stress, is an important indicator of a material’s ability to endure cyclic loading over time, particularly relevant in the context of gears subjected to repetitive rotational motion and varying loads during operation. On the other hand, rolling contact fatigue strength assesses a material’s resistance to failure under repeated contact and rolling action, which is typical in gear applications. This property is vital for evaluating a material’s endurance against wear, pitting, and surface distress resulting from the cyclic loading associated with rolling contact.

To ensure that heat-treated PA6 material exhibits comparable lifespan and performance to untreated PA6 in gear applications, it is essential to achieve the specified minimum values in Table 11.

In terms of wear, the iterative calculations are presented in Table 8 and Table 9, as well as in Figure 17. Figure 14 shows a comparison of the results for contact pressure; the differences between the obtained values are up to 3%.

The calculations in Table 8 and Table 9 contribute to the assessment of wear characteristics in the analysed gear system. The iterative approach involves successive calculations, refining the results based on the previous iteration. This method allows for a detailed understanding of the wear behaviour and enables the identification of influential factors.

Figure 17 presents a graphical representation of the wear results, enhancing the analysis with a visual dimension. The use of visual data assists in interpreting trends and patterns, improving the overall understanding of wear mechanisms.

To verify the accuracy of the simulations, Figure 14 compares the contact pressure results. As contact pressure significantly affects wear, the close alignment between simulated and actual values (with differences limited to 3%) demonstrates the reliability of the iterative calculations and the robustness of the wear analysis. This meticulous approach instils a high degree of confidence in the predicted wear behaviour, thereby enhancing the overall credibility of the study.

### 4.2. Correlation Between Mechanical Properties and Contact Pressure

A critical issue discussed in the previous section is the relationship between mechanical properties and contact pressure on tooth flanks during gear operation.

Through detailed analyses, the study aims to provide a concise and accurate description of the experimental results, their interpretation and the experimental conclusions that can be drawn, in order to provide a nuanced understanding of the complex relationship between material behaviour and the requirements of practical applications.

The correlation between the mechanical properties and the contact pressure, in particular in the context of cylindrical gears made of polyamide PA6 and subjected to heat treatments, leads to a series of considerations that are presented below.

- A direct correlation between the increase in modulus of elasticity (E) due to heat treatment and high contact pressures is established in the text.
- There is theoretical support for this correlation in Equation (1) and empirical support in the results shown in Table 7.
- The effect of heat treatment on root stress and contact pressure is shown by analytical methods, including stress calculations (Table 6 and Table 7).
- Root bending stress was found to have a relatively small effect on overall gear mechanical behaviour. This highlights the importance of the contact pressure on the flanks.
- The fatigue properties of the gear, as affected by heat treatment, are important for gear life and wear.
- Despite varying Young’s modulus, gears subjected to different heat treatments show a constant life, highlighting the influence of factors such as wear and related mechanisms, which are influenced by contact pressure and load cycles.
- It also highlights the importance of achieving the minimum levels of pulse and rolling contact resistance (Table 10) through heat treatment to ensure performance comparable to untreated PA6.
- Iterative calculations (Table 8 and Table 9) and graphical representation (Figure 17) of the wear results demonstrate the effect of contact pressure on wear phenomena.
- Comparison of simulated and actual contact pressure values provides validation and ensures the reliability of wear analysis.

In summary, the analysis provides a comprehensive investigation of the correlation between heat treatments, mechanical properties (in particular modulus of elasticity) and contact pressure in the context of cylindrical gears made from polyamide PA6. The emphasis of the study is on the practical implications of these findings for the optimisation of gear design and material treatments.

## 5. Implications and Applications

The implications of the research on the broader view of plastics behaviour in engineering applications are discussed, highlighting the potential for improving manufacturing processes and material performance. The contribution of the study to the optimization of PA6 for critical applications through FEA simulations is also highlighted, providing a pathway for the development of high-performance components without the need for physical realisation of the parts [33].

The research systematically analyses the results, providing a detailed understanding of how implementing heat treatments and modifying stress parameters in the manufacture of PA6 polyamide cylindrical gears can directly influence and improve mechanical properties, thus expanding the use of engineering plastics. In particular, the research identifies subtle changes in material properties, including mechanical strength, toughness, stiffness, wear behaviour and deformation resistance, and provides an innovative route to manufacturing processes and improving the overall performance of engineering plastics, particularly in the context of cylindrical gear applications.

These polymer materials are therefore suitable for constructing technical parts that can replace metal or for specific applications that must withstand extreme stresses.

In more general mechanical engineering applications, the research will have an impact where gears play a key role. Industries involved in mechanical engineering, from small appliances to large equipment, can benefit from the improved material properties.

To summarise, the research extends its application to industries that rely heavily on gears and provides a way to improve the overall performance, durability and efficiency of engineering plastics, particularly polyamide PA6, in a variety of engineering applications.

Due to their low material and manufacturing costs, polymer gears are being used in an increasing number of applications. They also offer a number of advantages that steel gears do not, in that they can be used without the need for lubricants.

PA6 gears are successfully used in office machinery for printer parts, household appliances for small motors, textile machinery, food processing and automotive industries, and due to their advantages of light weight, quiet running, corrosion resistance, low coefficients of friction, ease of mass production and ability to run without external lubrication.

## 6. Conclusions and Future Directions

The present study’s primary contribution is its focus on the impact of heat treatments on PA6 material and the enhancement of mechanical performance in cylindrical gears. This study provides a comprehensive and systematic investigation of the effects of heat treatments on PA6 material properties. It employs both numerical simulations with Ansys 2023 and analytical evaluations in accordance with the VDI 2736-2 code with KISSsoft software. This highlights the direct link between heat treatments, Young’s modulus and contact pressure, emphasising the importance of these factors on stress distribution, particularly in terms of root and tooth contact stresses. This approach not only facilitates the enhancement of PA6 gear design but also proposes a standardised methodological model that can be utilised in industrial applications. It thereby underscores the significance of a multidimensional approach in optimising the durability and practical performance of heat-treated materials.

This study demonstrated that heat treatments improve the mechanical performance of 3D-printed PA6 gears, but dimensional stability and wear must be carefully controlled, and the key findings include the following:

- Printing parameters strongly influence gear precision.
- Heat treatment enhances stiffness but may increase flank wear at higher temperatures.
- Anisotropy of printed PA6 should be explicitly modelled in future FEA.
- Dynamic load and thermal effects remain crucial areas for further study.
- Compared to conventional gears, 3D-printed PA6 gears offer lightweight, cost-effective, and rapid fabrication benefits, but with limitations in load capacity and thermal resistance.

The principal findings underscore the efficacy of heat treatments in enhancing the strength, stiffness and resilience of PA6 polymers, rendering them considerably more robust for gear applications. A significant aspect of this study is stress grading, which provides valuable insight into factors such as material fatigue, wear and contact pressure that influence the performance of heat-treated PA6 gears. The research establishes a clear relationship between heat treatments through Young’s modulus and contact pressure, thereby emphasising the importance of understanding the material behaviour of stress distribution, particularly in terms of tooth root and contact stresses. Based on the test results and simulations, it can be stated that, in the contact area of the wheels, on the flank of the teeth and closer to their base (above the inflection point of the tooth profile), wear occurs in the form of a crater (the depth of the formed “spot” has the maximum value).

This study underscores the necessity of attaining specified minimum levels of fatigue resistance under pulsating stress and rolling contact fatigue resistance through heat treatments to guarantee comparable longevity and performance to that of untreated PA6. By evaluating wear characteristics through iterative calculations and visual representations, this research offers insights into optimising gear design and material treatments. In conclusion, the findings contribute to a nuanced understanding of gear performance and durability, emphasising the importance of a multidimensional approach in improving the practical applicability of heat-treated PA6 in gear applications.

Among the heat treatment conditions investigated, annealing at 80 °C for 4 h provided the most balanced improvement in hardness and wear resistance while maintaining acceptable toughness and dimensional stability.

Some trade-offs were observed, such as treatments that improved wear resistance, which slightly reduced toughness; these insights highlight the need to tailor heat treatment parameters to the desired performance criteria.

These results provide actionable guidance for engineers, suggesting that material selection and heat treatment strategies can be optimized depending on the specific operational requirements of PA6 gears.

Overall, the study demonstrates that targeted heat treatments can significantly enhance gear performance, offering a practical framework for improving durability and reliability in industrial applications.

## Figures and Tables

**Table 1:** Additive printing parameters used to print PA6 gears—filament 3d printing manufacturer recommendation. (Source: authors based on the research course).

Parameter	Value
Nozzle thickness	0.8 mm
Print height layer	0.2 mm
Temperature of deposition bed	80 °C
Melt temperature	260 °C
Percentage of filling	100%

**Table 2:** The geometric parameters of the PA6 polymer gear samples were tested. (Source: authors based on the gear design).

Parameters	Symbol	Values
Number of teeth	*z*	37
Facewidth, mm	*b*	17
Normal module, mm	*m*	1.5
Normal pressure angle, °	α	20
Material	Own Input	PA6 (VDI 2736)
Accuracy grade in accordance with ISO1328:2020		A6
Reference diameter, mm	*d*	55.43
Tip diameter, mm	*d_a_*	59.56
Root diameter, mm	*d_f_*	52.08
Addendum coefficient	*haP*	1.00
Dendum coefficient	*hfP*	1.25
Centre distance, mm	*a*	56

**Table 4:** Comparative results between the approached methods (FEA-analytical) for the bending test. (Source: authors based on the finite element analysis results).

Bending Stress, MPa				
KISSsoft	Lewis Bending Equation	Classical Beam Theory	FEA	Finite Element Size, mm
14.65 Analytical base on VDI 2736 Part 2, [11]	11.14 $\sigma =\frac{F^{t}}{w\cdot m\cdot Y}$	14.16 $\sigma =\frac{F^{t}\cdot l\cdot t}{2\cdot I\cdot S_{cf}}$	9.25	0.25
			10.98	0.22
			11.53	0.18
			14.9	0.1

**Table 5:** Bending stress evaluation acco Abrasive wear is a critical consideration, especially in scenarios involving dry running conditions for cylindrical gears. The absence of lubrication exacerbates the challenges associated with abrasive wear in gear systems. Solid contaminants such as dust and particulate matter have a more direct and pronounced impact on the contacting surfaces of the gear teeth in the rding to VDI 2736-2 [11,25,26]. (Source: authors based on the bibliography).

Stress Type	Mathematical Expression [11,26]	Coefficients Used [11,26]	Meaning of the Coefficients [26]
**Ending stress**	$\sigma =K_{F}\cdot Y_{F_{a}}\cdot Y_{S_{a}}\cdot Y_{\varepsilon }\cdot Y_{\beta }\cdot \frac{F_{t}}{b\cdot m_{n}}$, MPa	$k_{F}=k_{A}\cdot k_{v}\cdot k_{FB}\cdot k_{F_{a}}$	$k_{F}—$tooth root load factor $k_{A}$—application factor $k_{v}$—dynamic factor $k_{FB}$—face load factor $k_{F_{a}}$—transverse load factor
		$F_{t}=\frac{2000\cdot T}{d}$	$F_{t}$—nominal tangential load, N *T*—nominal torque of pinion, N∙mm *d*—reference circle of pinion, mm
		$Y_{F_{a}}=\frac{\frac{6\cdot h_{Fa}}{m_{n}}\cdot cos\alpha_{Fan}}{{\left(\frac{S_{Fn}}{m_{n}}\right)}^{2}\cdot cos\alpha_{n}}$	$Y_{F_{a}}$—tooth form factor $m_{n}$—normal module, mm $\alpha_{Fan}—$pressure angle $\alpha_{n}—$normal pressure angle $h_{Fa}$—bending moment arm relevant to load application at the tooth tip, mm
		$Y_{s_{a}}=\left(1.2+0.13\cdot L_{a}\right)\cdot q_{s}^{\left(\frac{1}{1.21+2.3/L_{a}}\right)}$ $L_{a}=S_{Fn}∕h_{F_{a}}$	$Y_{S_{a}}$—stress correction factor $q_{s}—$notch factor $S_{Fn}—$Tooth root chord at the critical section, mm
		$Y_{\varepsilon }=0.25+\frac{0.75}{\varepsilon_{a}}$	$Y_{\epsilon }$—contact ratio $\varepsilon_{a}$—transverse contact ratio
		$Y_{\beta }=1-\varepsilon_{\beta }\frac{\beta }{120{}^{\circ}}$	$Y_{\beta }$—helix angle factor $\varepsilon_{\beta }$—overlap ratio β—helix angle
		$Y_{ST}=2.0$	$Y_{ST}—$stress correction factor
**Permissible** **bending stress**	$\sigma_{\mathrm{FG}}=\sigma_{F_{\lim}}\cdot Y_{\mathrm{NT}}\cdot Y_{\mathrm{ST}}$ $\sigma_{\mathrm{Fp}}=\frac{\sigma_{FG}}{S_{Fmin}}$, MPa $\sigma_{FG}=Y_{ST}\cdot \sigma_{F_{\lim}}$		$\sigma_{\mathrm{Fp}}$—allowable stress on the tooth root, MPa $S_{Fmin}—$the required minimum safety factor for continuous operation is generally $S_{Fmin}\;$ = 2.0. $\sigma_{FG}—$maximum root strength, MPa
**Allowable bending stress**	$\sigma_{F_{\lim}}$ defined as stress level with 10% failure probability [26]		$\sigma_{F_{lim}\;}$—fatigue strength (nominal root stress), MPa [27]
**Service life factor**	$Y_{\mathrm{NT}}$ combined into $\sigma_{F_{\mathrm{limN}}}$ [26]		$Y_{NT}—$life factor

**Table 6:** Service life and bending stress calculation results. (Source: authors based on the calculation results).

Method	*H* Service Life, hr	*K_A_* Application Factor	*S_F_* Safety for Tooth Root Stress	$\;\sigma_{F_{0}}$ Nominal Stress at Tooth Root, MPa	$\;\sigma_{F}$ Tooth Root Stress, MPa	$\;\sigma_{F_{P}}$ Permissible Tooth Root Stress, MPa
PA6 Untreated (*E* = 2100 MPa)	3024	1.25	4.83	11.72	14.65 (14.14 FEA)	54.46
PA6 160 °C annealing temperature (*E* = 6000 MPa)	3024	1.25	4.83	11.72	14.65 (14.31 FEA)	54.46

**Table 7:** Service life and flank stress calculation results (pitting). (Source: authors based on the calculation results).

Method	H, hr	*S_H_* Safety Factor for Contact Stress on Operating Pitch Circle	*σ_H0_* Nominal Contact Stress, MPa	*σ_H_* Contact Stress at Operating Pitch Circle, MPa	*σ_HP_* Permissible Contact Stress, MPa
PA6 Untreated (*E* = 2100 MPa)	3024	1.46	20.46	22.87 (20.887 FEA)	35.26
PA6 120 °C annealing temperature (*E* = 4000 MPa)	3024	1.06	28.23	31.57	35.26
PA6 140 °C annealing temperature (*E* = 5000 MPa)	2973	0.95	31.57	35.29	35.26
PA6 160 °C annealing temperature (*E* = 6000 MPa)	1024	0.87	34.58	38.66	35.26

**Table 8:** Wear results for non-iterative calculation (VDI 2736-2, KISSsoft). (Source: authors based on the software results).

PA6	Local Linear Wear, µm		Wear, Volume per Tooth, mm^3^	Wear, Mass per Gear, g	Hertzian Pressure, N/mm^2^
	Min	Max			
PA6—Untreated (*E* = 2100 MPa)	0.89	267	2.77	0.11	22.61
PA6—120 °C annealing temperature (*E* = 4000 MPa)	0.90	251	2.66	0.11	42
PA6—140 °C annealing temperature (*E* = 5000 MPa)	0.90	242	2.6	0.11	46
PA6—160 °C annealing temperature (*E* = 6000 MPa)	0.90	231	2.53	0.11	54

**Table 9:** Wear results for iterative calculation (VDI 2736-2, KISSsoft). (Source: authors based on the software results).

PA6	Local Linear Wear, µm	Wear, Volume per Tooth, mm^3^	Wear, Mass per Gear, g
PA6—Untreated (*E* = 2100 MPa)	264	3.93	0.164
PA6—120 °C annealing temperature (*E* = 4000 MPa)	280.74	4.48	0.187
PA6—140 °C annealing temperature (*E* = 5000 MPa)	271	4.54	0.19
PA6—160 °C annealing temperature (*E* = 6000 MPa)	403	5.95	0.25

**Table 10:** The validation of numerical analyses (regarding material loss due to wear) presented through the comparison of percentage differences relative to experimental simulations.

PA6	Wear, Mass per Gear—Iterative Calculation, g	Wear, Mass per Gear-Experimental Calculation, g	Percentage Difference, %
PA6—Untreated (*E* = 2100 MPa)	0.164	0.141	4.17
PA6—120 °C annealing temperature (*E* = 4000 MPa)	0.187	0.194	3.67
PA6—140 °C annealing temperature (*E* = 5000 MPa)	0.19	0.197	3.61
PA6—160 °C annealing temperature (*E* = 6000 MPa)	0.25	0.258	3.14

**Table 11:** Minimum values required for fatigue strength under pulsating stress (nominal stress) and rolling contact fatigue strength, which must be achieved through heat treatments applied to PA6. (Source: authors based on the test results).

Fatigue Strength Type	PA6 Non-Treated (*E* = 2100 MPa) (Retrieved from the KISSsoft Database)		PA6 Treated 120 °C Annealing Temperature		PA6 Treated 160 °C Annealing Temperature	
	600 h	3000 h	600 h	3000 h	600 h	3000 h
Fatigue strength under pulsating stress (nominal stress), MPa	43.4	35.4	44	38	48	43
Rolling contact fatigue strength, MPa	41.9	33.5	44	38	48	43

## Data Availability

The original contributions presented in this study are included in the article. Further inquiries can be directed to the corresponding authors.
